# Dandy-Walker syndrome: an updated literature review

**DOI:** 10.1007/s00381-025-06842-0

**Published:** 2025-05-30

**Authors:** Maria Isabel Ocampo-Navia, Wilfran Perez-Mendez, Maria Paula Rodriguez-Alvarez, Juan Chadid-Contreras, Manuel Francisco Vergara

**Affiliations:** 1 Department of Neurosurgery, Pontificia Universidad Javeriana, Bogotá, Colombia; 2 Department of Neurosurgery, Hospital Universitario San Ignacio, Bogotá, Colombia; 3 Neurosurgery Research Group, Facultad de Medicina, Pontificia Universidad Javeriana, Bogotá, Colombia

**Keywords:** Dandy-Walker syndrome, Dandy-Walker malformation, Blake’s pouch cyst, Mega cisterna magna, Hydrocephalus

## Abstract

The Dandy-Walker syndrome (DWS) encompasses a group of anatomical midline cerebellar disorders with potential shared embryological origins, including the classic Dandy-Walker malformation, Blake’s pouch cyst, and mega cisterna magna. Genetic factors, chromosomal abnormalities, and environmental influences contribute to its etiology. DWS, occurring in 1 in 25,000 to 35,000 live births, often presents with hydrocephalus and other central nervous system anomalies. Clinical manifestations vary, with symptoms appearing from neonatal to adult stages. Diagnosis is performed through neuroimaging, evaluating the posterior fossa and associated anomalies. Management involves treating hydrocephalus, addressing associated anomalies, and providing neurological follow-up with a multidisciplinary team. Prognosis hinges on associated malformations and their severity, impacting long-term outcomes. An unsystematic updated review on the embryology, pathophysiology, diagnostic approach, and therapeutic management of DWS is presented.

## Introduction

Dandy-Walker syndrome (DWS) is a heterogeneous group of anatomical disorders, potentially stemming from a shared embryological etiology, characterized by abnormalities in the development of midline cerebellar anatomy. These anomalies range from hypoplasia to agenesis, or even early cerebellar atrophy associated with a large cyst in the posterior fossa, typically in communication with the fourth ventricle [1]. Within this spectrum of pathologies are the classic Dandy-Walker malformation (DWM), Blake’s pouch cyst (BPC), and mega cisterna magna (MCM) [2]. It is important to acknowledge the historical use of the term “Dandy-Walker variant”, which has been discouraged in recent years due to its lack of specificity and its inability to adequately differentiate between distinct pathologies with similar imaging findings, such as inferior vermian hypoplasia (IVH) and other neurogenetic conditions that result in an enlarged retrocerebellar cerebrospinal fluid (CSF) space [3].

The first post-mortem description of this condition was made by Sutton in 1887 [4]. Later, in 1914, Dandy and Blackfan reported the presence of hydrocephalus, an enlarged posterior fossa, agenesis of the cerebellar vermis, and cystic dilation of the fourth ventricle in a 13-month-old infant, and these findings were subsequently further characterized by Walker in 1921 [5–7]. The term “Dandy-Walker Malformation” was first introduced by Benda in 1954, who described six cases exhibiting the features initially reported by Dandy [8]. Benda hypothesized that abnormalities in the normal regression of the posterior medullary velum led to the formation of cystic dilations in the fourth ventricle [8].

The estimated annual incidence is 1 in every 25,000 to 35,000 live births, with a male-to-female ratio of 1:3. Hydrocephalus occurs in approximately 80% of DWS cases and accounts for 4 to 12% of pediatric hydrocephalus cases [9]. The aim of this article is to provide an updated literature review of the embryology, pathogenesis, clinical manifestations, diagnosis, and treatment of the DWS.

## Definitions

### Dandy-Walker malformation (DWM)

DWM has been defined by a series of imaging features that can be clearly identified on mid-sagittal ultra-thin T2-weighted images: (1) cystic dilation of the posterior fossa extensively communicating with the fourth ventricle; (2) complete or partial agenesis of the lower portion of the vermis in varying degrees (lower three-fourths, lower half, lower one-fourth); (3) hypoplasia, anterior rotation, and upward displacement of the remaining vermis; (4) absence or flattening of the fastigium angle; (5) enlargement of the posterior fossa with upward displacement of the transverse sinuses, tentorium, and torcula; and (6) anterolateral displacement of the cerebellar hemispheres [10]. Hydrocephalus occurs in up to 80% of cases but is not considered a defining feature of DWM [11]. Although this definition has been used for many years to describe DWS, we will later see that the imaging diagnostic criteria have changed. While the enlargement of the posterior fossa was previously considered a key criterion, recent studies have demonstrated that this is no longer the case [12].

### Dandy-Walker complex (DWC)

Comprises a spectrum of anomalies of the posterior fossa, classified from mild (MCM only or persistent Blake’s pouch) to moderate (enlargement of the fourth ventricle and mild vermis hypoplasia) and to severe (agenesis of the vermis and cystic dilation of the posterior fossa communicating with the fourth ventricle) [13, 14].

### Mega cisterna magna

The term MCM was first introduced by neurosurgeon Gonsette in 1962 to describe an enlargement of the cisterna magna, initially defined by ventriculographic measurements exceeding 15 mm in length, 5 mm in height, and 20 mm in width [15]. Currently, MCM is defined as a retro- and infracerebellar cerebrospinal fluid (CSF) space greater than 10 mm on midsagittal images, with an intact cerebellar vermis, a normal fourth ventricle, and a regular torcular location [16]. Unlike other posterior fossa malformations, MCM freely communicates with the fourth ventricle and is not associated with hydrocephalus.

### Persistent Blake’s pouch

BPC, also known as persistent Blake’s pouch (PBP), manifests as a cystic collection, located inferior and posterior to the cerebellum [2]. In this condition, there is no free communication between the fourth ventricle and the surrounding subarachnoid space in the midline. Due to this, some authors consider that Blake’s pouch cyst does not fall within the Dandy-Walker spectrum [11]. The cerebellar vermis and cerebellum have a normal morphology. Characteristically, the choroid plexus curves beneath the cerebellar vermis to position itself within the cyst’s upper part [17].

## Etiology

DWS is a group of medical conditions that are influenced by various factors that modify and shape the progression of symptoms, manifestations, and its numerous variations. The existing literature underscores the presence of an etiologic heterogeneity, prompting the need for a tailored approach to diagnosis and treatment for each patient [18]. Among the various causal factors, chromosomal abnormalities emerge as prevalent drivers behind the emergence of both DWM and DWV. Additionally, the influence of epigenetic mechanisms and environmental variables, such as maternal alcohol consumption, gestational diabetes, and infections during pregnancy, significantly contributes to the intricate development of the malformation [9].

Currently, the study of the genetic bases that are involved in the DWS has shown the implication of genes such as ZIC1 and ZIC4 [19]. The molecular pathways contributing to these phenotypes are not fully explored; nevertheless, it has been demonstrated that *Zic* genes are key in neural development [20]. This gene expression initiates prior to the appearance of the cerebellar primordium and can be traced back to neuroectoderm formation at the earliest point of neural development [21, 22].

Most research concerning these molecular pathways has been done in mice; however, evidence in humans strongly shows the expression of ZIC1 and other ZIC genes at the inner granule cell layer of the cerebellum. In 2009, three genes were described in association with DWM: FOXC1 on human chromosome 6p25, and the linked ZIC1 and ZIC4 genes on human chromosome 3q24 [23]. One year later, it was reported that the diminished cerebellar dimensions observed in ZIC1 and ZIC4 mutants can be attributed to a reduction in postnatal granule cell progenitor proliferation. This finding corroborates the indispensable role of these genes in cerebellar development and offers a valuable model for elucidating the developmental mechanisms underlying this clinically significant congenital malformation [24].

## Embryology and pathogenesis

DWC is the result of rhombencephalon and fourth ventricle dysembryogenesis. Understanding the embryonic development of the brainstem and cerebellum is important to comprehend the pathogenesis of different cystic anomalies in the posterior fossa [17].

### Formation of the primary brain vesicles

In the fourth week of gestation, the cranial neuropore closes, and the three primary brain dilations are established: the prosencephalon (forebrain), the mesencephalon (midbrain), and the rhombencephalon (hindbrain). After the closure of the neural tube, the neural canal located in the posterior region of the rhombencephalon will form the fourth ventricle [11].

The rhombencephalon will subsequently divide into two regions through the pontine fold: the metencephalon, which leads to the development of the cerebellum and the pons, and the myelencephalon, which will develop into the medulla oblongata [25]. As the pontine fold continues to extend, it leads to the widening and thinning of the roof plate of the fourth ventricle and the separation of the lateral walls of the rhombencephalon, conferring its rhomboid shape [17].

**Fig. 1:**
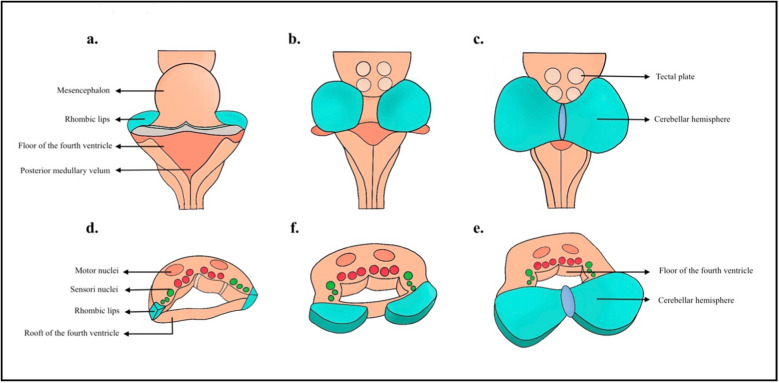
The diagram of the brainstem illustrates the growth sequence of the cerebellum from the rhombic lips (blue). These structures develop along the lateral edges of the rhombencephalon and serve as the site for cerebellar differentiation

In the sixth week of development, there is bilateral thickening at the lateral edges of the roof plate of the fourth ventricle, known as rhombic lips. These gradually expand to shape the cerebellar hemispheres, which, by the 9 th week of gestation, fuse at the midline in a cranial-to-caudal direction. Along with the dorsal alar plate of the mesencephalon, they form the cerebellar vermis [25] (Fig. 1).

**Fig. 2:**
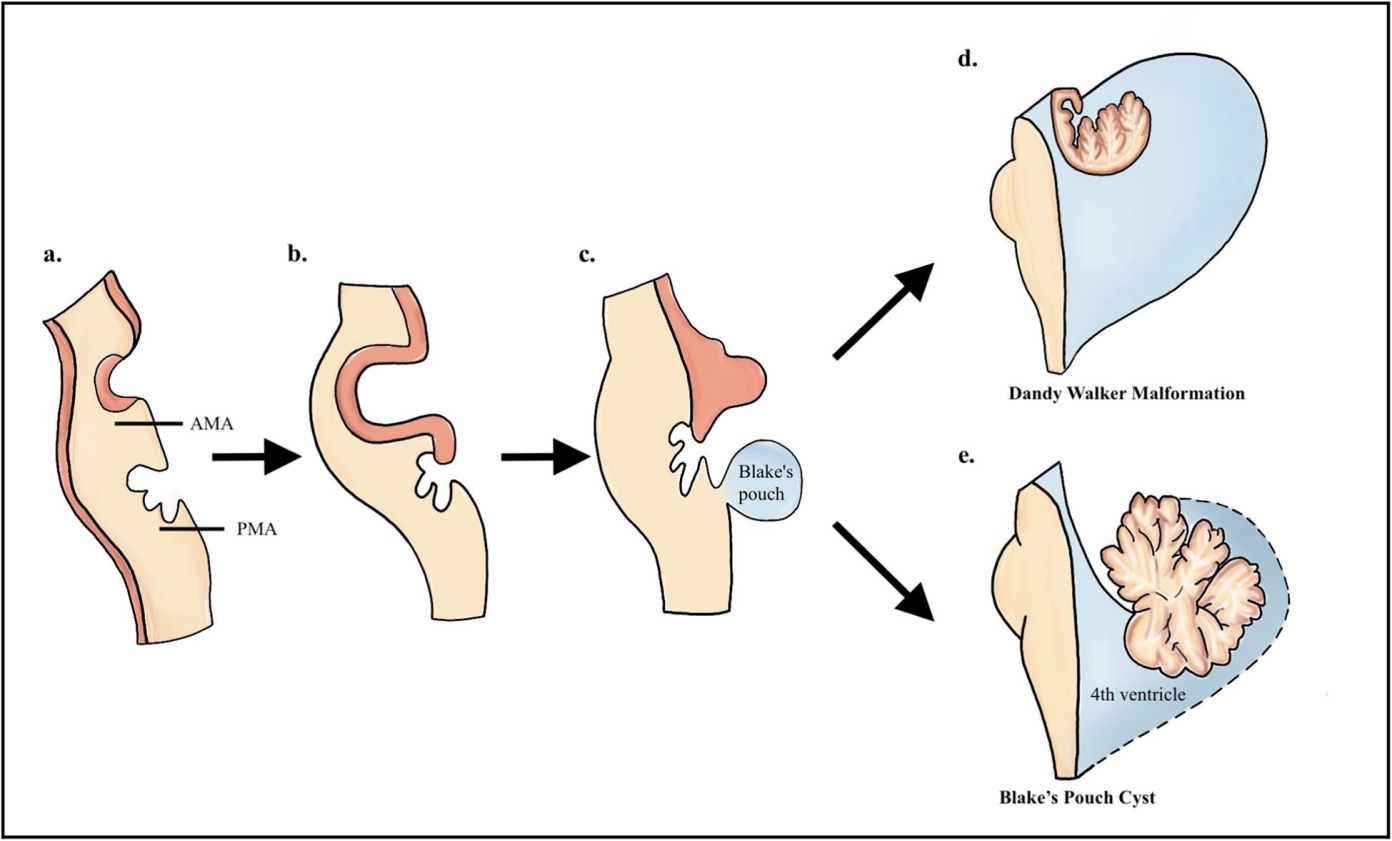
Schematic illustration of normal fourth ventricle development in sagittal views. **a**–**c** During the tenth week, the choroid plexus precursor compresses the roof plate, forming the plica choroidalis, which divides it into the anterior membranous area (AMA) and posterior membranous area (PMA). The growth of the rhombic lips pushes the AMA caudally, while the PMA fills with CSF, forming the Blake’s pouch, which later gives rise to the median foramen of Magendie. **d** Representation of Dandy-Walker malformation caused by the failure of AMA integration with the choroid plexus. **e** Representation of Blake’s pouch cyst caused by the failure of PMA regression

In the tenth week, the tissue that will develop into the choroid plexus presses against the roof plate of the fourth ventricle. This indentation is known as the plica choroidalis and divides the roof plate into an upper portion called the anterior membranous area (AMA) and a lower portion known as the posterior membranous area (PMA) [25, 26]. As the rhombic lips grow, they push the AMA of the plica choroidalis caudally, which later decreases in size and fuses with the choroid plexus [11]. The PMA fills with cerebrospinal fluid (CSF) to form the “Blake’s pouch,” a closed cavity that does not communicate with the subarachnoid space of the cisterna magna. Later on, Blake’s pouch becomes permeable to give rise to the median foramen of Magendie [11, 17] (Fig. 2a–c).

### Dandy-Walker malformation

**Fig. 3:**
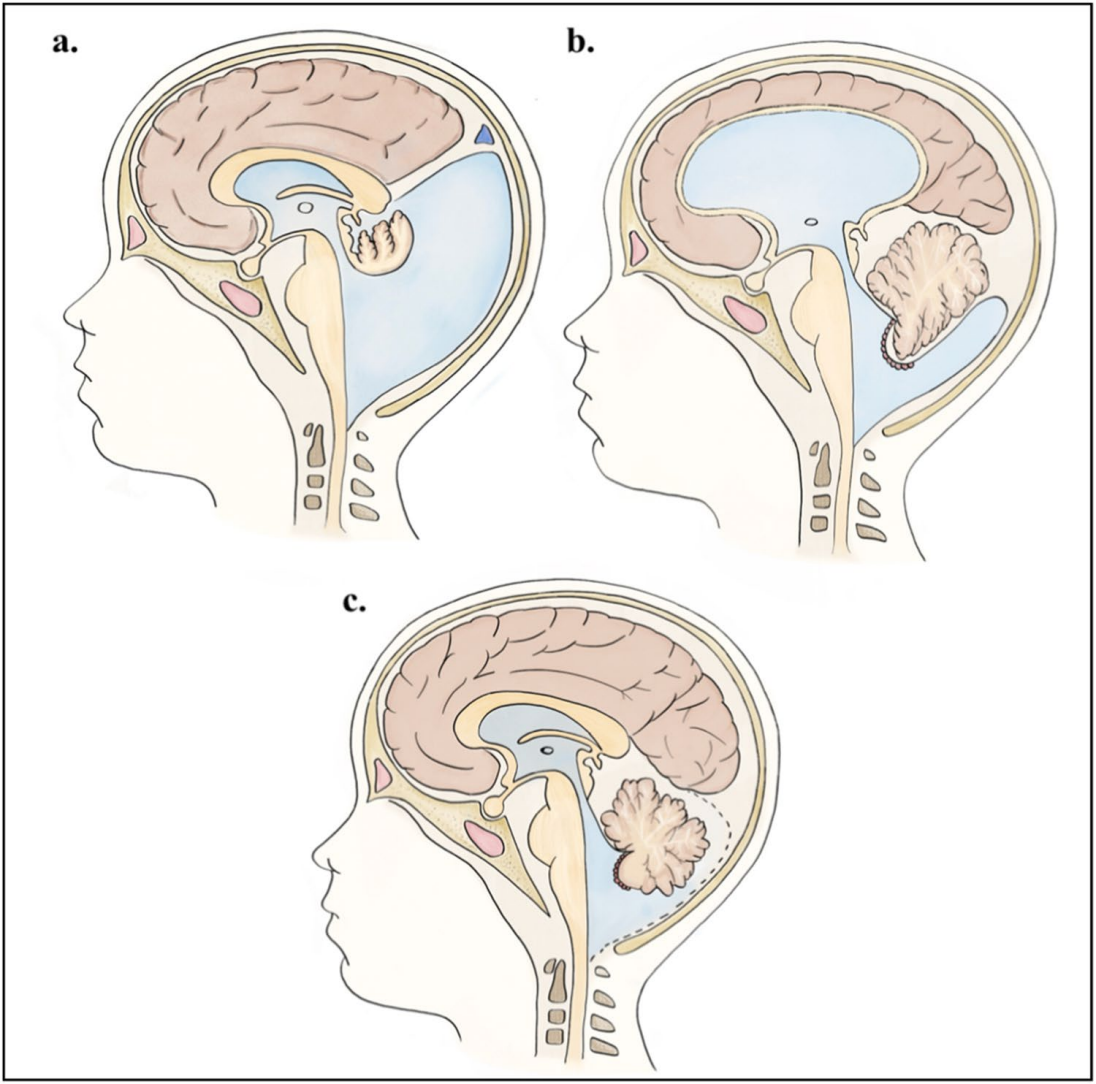
Schematic illustration of Dandy-Walker syndrome. **a** Dandy-Walker malformation. **b** Blake’s pouch cyst. **c** Mega cisterna magna

DWM results from a failure in the integration of the AMA with the choroid plexus [11]. The pulsations of CSF cause the AMA to expand into the posterior region of the posterior fossa, located between the caudal edge of the developing vermis and the cranial edge of the choroid plexus. This leads to the formation of a large posterior cyst, displacing the embryonic vermis cranially, altering its development, and resulting in complete or partial agenesis of the vermis, associated with a counterclockwise rotation of this structure [25]. This cystic formation is often covered by a membrane that blocks communication with the subarachnoid space; however, in some cases, the permeabilization of the AMA occurs, which may explain the presence or absence of hydrocephalus in this condition. Additionally, it is suggested that the enlargement of the posterior fossa is secondary to arrested development of the tentorium, straight sinus, and torcular Herophili, caused by the abnormal dilatation of the fourth ventricle [11, 17] (Fig. 3a).

### Blake’s pouch cyst

BPC relates to an anomaly in the development of the PMA, which is attributed to a failure in the regression of the PMA and subsequent fenestration in the foramen of Magendie. This results in the formation of a cystic collection, located inferior and posterior to the cerebellum, causing diffuse enlargement of the fourth and supratentorial ventricles [27, 28] (Fig. 2). This enlargement cannot be compensated for by the consequent opening of the Luschka foramina and leads to the development of tetraventricular hydrocephalus [17] (Fig. 3b).

### Mega cisterna magna

MCM is a condition characterized by an abnormal enlargement of the cisterna magna. It is due to a late permeabilization of Blake’s pouch, leading to its enlargement and expansion of the posterior fossa before its permeabilization [29]. In this condition, there is free communication of CSF between the fourth ventricle and the surrounding subarachnoid space [30]. It is distinguished by the normal appearance of the cerebellar vermis and hemispheres, as well as the absence of hydrocephalus and the lack of a mass effect on the surrounding cerebellum and cerebellar tentorium [17] (Fig. 3c).

## Clinical manifestations

The signs and symptoms present in DWS depend on the type of structural anomaly, the severity of the condition, associated malformations, and the age at diagnosis. In numerous cases, the diagnosis is already established through prenatal studies. However, in milder cases, the diagnosis may not be made until late adolescence [1].

Approximately 80% of DWM patients have hydrocephalus. Although hydrocephalus is an associated complication rather than a component of the malformation itself, it is often diagnosed before 3 months of age [17, 25]. Mega cisterna magna is often asymptomatic and typically detected incidentally [17].

The clinical characteristics resulting from increased intracranial pressure are influenced by both the patient’s age and the severity of hydrocephalus [31]. In the pediatric population, the most common postnatal sign of increased intracranial pressure is the progressive increase in head circumference or macrocephaly [32]. Other signs include the presence of downward deviation of the eyeballs associated with retraction of the upper eyelids (setting sun sign), paralysis of upward vertical gaze, bulging of the anterior fontanelle, diastasis of the cranial sutures, and dilation and congestion of the scalp veins [1].

Once the closure of fontanelles is completed in older children, the clinical manifestations are similar to those observed in patients with cerebellar tumors. Key symptoms include ataxia, nystagmus, spasticity, and impaired fine motor control [33]. Conversely, sporadic cases of asymptomatic adults with variants of the DWS have been documented, sometimes exclusively associated with cognitive disorders or psychiatric conditions, such as schizophrenia or bipolar disorder [34, 35].

## Associated malformations

DWM is frequently associated with multiple intracranial and extracranial anomalies, which significantly impact the disorder’s outcome and the severity of clinical symptoms. Postnatal studies have reported that associated malformations occur in 50 to 70% of cases. The severity of these disorders can vary widely, from severe clinical presentations to almost asymptomatic cases. These malformations may be categorized as neurologic, systemic, or genetic and often coexist. Among the most common central nervous system (CNS) anomalies linked to DWM are ventriculomegaly, holoprosencephaly, encephalocele, and agenesis of the corpus callosum [9].

Typically, the initial symptoms of DWM are related to the CNS, such as hydrocephalus. However, the diagnosis of DWM may also occur due to the presence of systemic symptoms, including cardiovascular anomalies like transposition of the great arteries and congenital pulmonary stenosis; urogenital conditions such as hydrocele and horseshoe kidney; intestinal abnormalities like duodenal atresia, megarectum, and megasigmoid; and craniofacial anomalies including cleft palate, strabismus, and facial angiomas. Additionally, limb malformations and syndactyly of the fingers or toes have also been reported in association with DWM [31].

Recent studies suggest a potential association between posterior fossa abnormalities, such as DWS and psychiatric symptoms. However, the existence of a causal relationship remains unclear. Cognitive disabilities and epileptic seizures may be observed in a subset of patients, particularly in children older than 1 year [36].

## Diagnosis

### Prenatal diagnosis

The initial approach to these anomalies should be conducted using ultrasound, which is the preferred imaging technique for prenatal diagnosis [9]. It is crucial to make the diagnosis starting from the 18 th week of gestation because it has been previously confirmed that the cerebellar vermis completes its development around the 17 th to 18 th weeks. In weeks 15 to 16, it is expected that the cerebellar vermis may not be fully formed, so an early diagnosis could lead to false positives [37].

To diagnose DWM, it is necessary to visualize both the axial and sagittal planes of the posterior fossa [38]. In the mid-sagittal plane, the vermis should be identified, and its appearance and dimensions should be assessed, including its vertical diameter, which is particularly pertinent because most cases of vermis defects entail caudal portion agenesis [37]. A vermis that appears normal but is notably small suggests vermian hypoplasia. A small vermis lacking the fastigium, fissure, or both points to partial vermian agenesis [9].

In addition, it is important to check for any upward displacement of the tentorium and torcula, as some authors suggest that cerebellar tentorium displacement is a fundamental criterion for the differential diagnosis of these anomalies [39]. In DWM, the tentorium is completely displaced upwards; however, in BPC or arachnoid cyst, a focal displacement may occur as a result of mass effect [40].

Moreover, another feature to discriminate PF anomalies includes measuring the brainstem-vermis (BV) angle, which is obtained by drawing a tangent line to the dorsal surface of the brainstem and a second line tangential to the ventral contour of the vermis [9]. This measurement allows for the categorization of the upward rotation of the vermis. After 20 weeks’ gestation BV angles exceeding 45° suggest DWM, whereas angles below 30° imply a BPC [40].

Although ultrasound is the primary technique for prenatal diagnosis, detailed anatomical delineation and definitive radiological characterization are essential. In some cases, sonographic imaging has limitations; therefore, magnetic resonance imaging (MRI) should be considered [25, 40]. It has been proposed that the clinical features described in the definition of DWM are indicative and necessary for its diagnosis [40].

Additionally, Nagaraj et al. proposed that the tegmentovermian angle (TVA) serves as a valuable tool for differentiating classic DWM from other posterior fossa abnormalities. In their study, TVA was markedly larger in DWM compared to the vermian hypoplasia and Blake’s pouch remnant groups, regardless of gestational age, with measurements ranging from 79° to 130° [41].

On the other hand, MRI findings vary for each pathology and are detailed further in Table 1.

**Table 1:** Diagnostic findings of posterior fossa anomalies in the fetus (adapted from Robinson AJ, et al., 2016)

**Diagnosis**	**Vermis**		**Blake´s pouch**	**Posterior fossa**	**Torcula**	**Choroid plexus position**	**Hydrocephalus**
	**Tegmentovermian angle**	**Hypoplasia**					
**Dandy-Walker malformation**	Yes, > 40–45°	Yes, variable, may be severe	Enlarged	Enlarged	Elevated	Inferior margin of Blake’s pouch	Yes (in 80% of cases)
**Blake’s pouch cyst**	Yes, mild to moderate (< 30°)	No	Enlarged	Normal	Normal	Superior margin of Blake’s pouch	Yes
**Mega cisterna magna**	No	No	Enlarged	Enlarged	-	Superior margin of Blake’s pouch	No

### Postnatal diagnosis

**Fig. 4:**
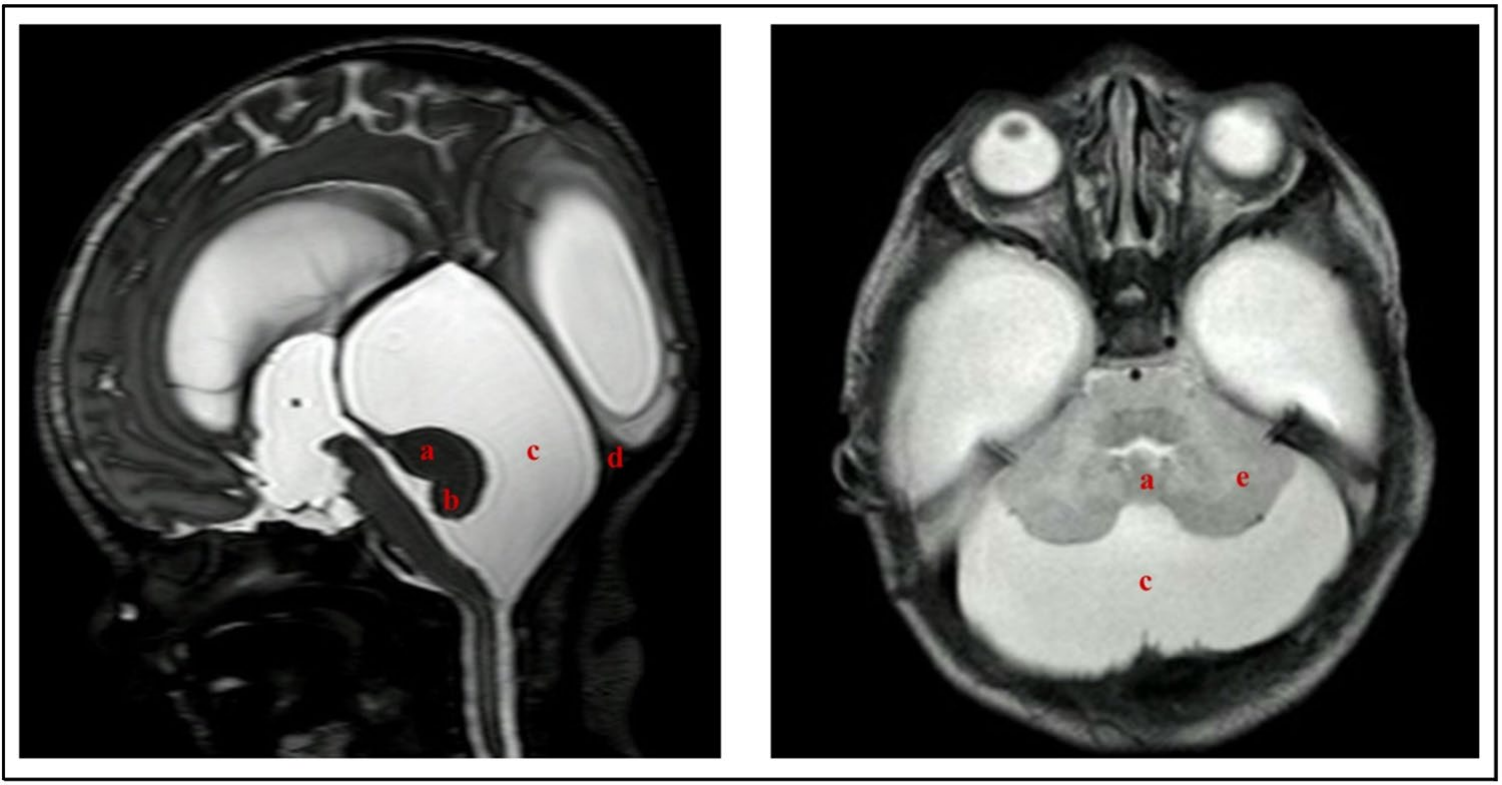
Postnatal sagittal (left) and axial (right) T2-weighted MRI of Dandy-Walker malformation. (a) Hypoplasia, anterior rotation, and superior displacement of the remaining portion of the vermis**.** (b) Agenesis of the inferior portion of the vermis. (c) Large posterior fossa cyst communicating with the fourth ventricle**.** (d) Upward displacement of the tentorium and torcula. (e) Anterolateral displacement of the cerebellar hemispheres. Note the large supratentorial ventricular dilation characteristic of hydrocephalus

Malformations detected during prenatal life should be confirmed through ultrasound and/or MRI after birth. Currently, MRI is considered the most accurate imaging tool for the diagnosis as it allows for a better characterization of anatomical features, as shown in Fig. 4 [33].

Whitehead et al. proposed updated imaging criteria for diagnosing DWM, emphasizing five key features: (1) hypoplasia predominantly affecting the inferior vermis, (2) unpaired caudal lobule, (3) obtuse fastigial recess, (4) large TVA, and (5) inferolateral displacement of the taenia-tela choroidea complex and choroid plexus [12]. Additionally, they recommended excluding posterior fossa size and torcular location from the diagnostic criteria, as these parameters are influenced by the degree of fourth ventricular outflow obstruction rather than being direct manifestations of the malformation. Furthermore, comparative imaging analysis of DWM and vermian hypoplasia (VH) demonstrated that, in DWM, the fastigial angle is typically flat or obtuse, whereas, in VH, it remains acute [12].

An additional imaging finding, the “tail sign,” is a radiological feature observed on MRI, characterized by a linear hypointensity on T2-weighted images at the inferior portion of the cerebellar vermis, resembling a “tail.” This finding is associated with thickening of the fourth ventricular roof and abnormal vermian morphology. Although its specificity remains debated, the tail sign has been proposed as a distinguishing feature in the differential diagnosis of DWM [12, 42, 43].

## Differential diagnosis

DWS must be distinguished from other cystic malformations of the posterior fossa, such as cerebellar hypoplasia, which is defined by a normal cerebellar vermis, with a transverse cerebellar diameter below the fifth percentile and arachnoid cysts, which can be located anywhere in the posterior fossa and do not communicate with the subarachnoid space or the surrounding ventricular system [40]. MRI is the imaging modality of choice for diagnosing arachnoid cysts, as it provides high-resolution visualization of their morphology, location, and mass effect on adjacent structures. Given its diagnostic accuracy, CT cisternography is rarely utilized in clinical practice [11, 44].

Other entities with overlapping imaging features include panventriculomegaly (PaVM), defined by a wide foramen of Magendie and a large cisterna magna, a congenital condition with tetraventricular dilation and possible genetic associations, such as *DNAH14* mutations [45]. Despite the presence of patent ventricular outlets, this condition may still benefit from endoscopic third ventriculostomy (ETV), as suggested by Al-Hakim et al. and Kehler et al., who proposed that an occult intracisternal obstruction or pressure gradient at the level of the third ventricle may underlie the pathophysiology ​[46, 47].

## Treatment

**Fig. 5:**
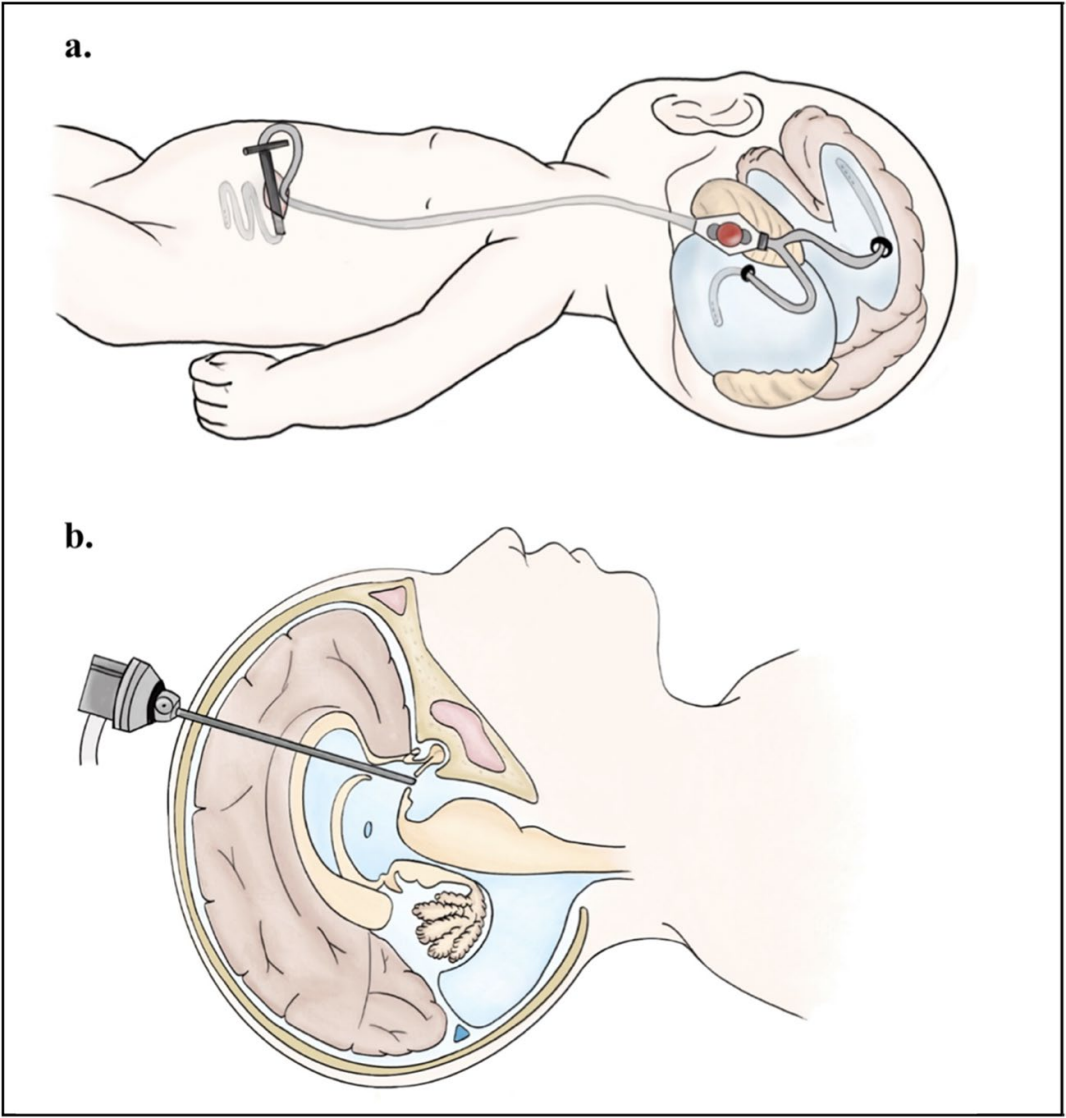
Surgery strategies for hydrocephalus in Dandy-Walker syndrome. **a** Cyst ventriculoperitoneal shunt. **b** Endoscopic third ventriculostomy

The therapeutic objectives for DWM are not aimed at correcting the primary CNS malformation, but rather at managing hydrocephalus and the posterior fossa cyst. Treatment options vary according to the severity of symptoms and the presence of associated anomalies [48]. The main strategies include surgical procedures such as cyst membrane fenestration, cystoperitoneal shunting, ventriculoperitoneal shunting, cyst-ventriculoperitoneal shunting, and endoscopic third ventriculostomy with or without choroid plexus cauterization [48–50] (Fig. 5).

Previously, the treatment of choice involved the fenestration or excision of posterior fossa membranes to facilitate CSF flow. However, this technique was later shown to have a high failure rate, as up to 75% of patients still required a shunt and mortality approached 10% [51, 52]. Currently, cerebrospinal fluid shunting is a commonly used option to manage associated hydrocephalus. However, there has been debate about the type of shunt to be placed. Several authors have supported the use of ventriculoperitoneal (VP) shunts due to their ease of placement and lower complication rates, such as malposition or migration [53]. Nevertheless, transtentorial ascending herniation and acquired aqueductal stenosis are risks associated with this procedure, leading many to initially opt for cystoperitoneal (CP) shunting [54].

Additionally, some authors have studied the outcomes in children with VP shunts compared to those with CP shunts, finding no significant differences in terms of intellectual development, frequency of shunt revisions, or complication rates [55, 56]. However, Bindal, et al. noted that 3 out of 21 patients who underwent CP shunting developed posterior fossa subdural hematomas, one of whom required a craniotomy for removal. Based on these findings, they suggested that CP shunts carry an additional risk that should be avoided and recommended VP shunting as the preferred initial management strategy [55].

On the other hand, the combined placement of cyst-ventriculoperitoneal (CPVP) shunts has been used to equalize pressure across the tentorium and reduce the risk of herniation, with high success rates. It has been reported that up to 38% of patients with a single shunt required the placement of an additional shunt. Consequently, single-shunt strategies may ultimately need a second shunt to prevent the expansion of the untreated compartment [55]**.** However, it has been suggested that CPVP shunting may cause secondary aqueductal stenosis due to the significant reduction of CSF flow through the aqueduct [57]. Therefore, several authors have proposed that, to reduce the risk of complications, the posterior fossa architecture of patients should be studied using high-resolution imaging before any surgical intervention, as this is associated with a better prognosis [48, 53].

Endoscopic third ventriculostomy (ETV) has been primarily used to treat obstructive hydrocephalus due to aqueductal stenosis, although its use for treating the DWM has also been documented [53, 57]. This allows for a more physiological treatment of CSF and reduces the infection rate by avoiding the use of foreign bodies. However, the anatomy of the third ventricle and the communication of the cyst with the supratentorial ventricular system must be favorable for performing ETV [58]. ETV has emerged as a reasonable initial treatment option for patients with DWM, particularly those with a patent aqueduct. If ETV fails, a VP or CP shunt may be considered [53].

Finally, the process of choosing the appropriate procedure is greatly influenced by the presence of aqueductal obstruction on preoperative neuroimaging. If obstruction is detected, it is necessary to drain the supra- and infratentorial compartments to avoid a transtentorial pressure gradient, making ETV a viable option as initial treatment, especially in patients with a patent aqueduct. In cases of aqueductal stenosis, aqueductoplasty or stent placement, in addition to ventriculostomy, is justified to ensure adequate communication between compartments [53].

## Prognosis

In recent years, research has underscored the crucial impact of accurate fetal diagnosis, the detection of associated abnormalities, on the long-term prognosis of patients with DWS. According to the existing literature, infants born with congenital defects affecting two or more organ systems tend to exhibit the poorest survival rates [40]. Nevertheless, despite numerous contemporary studies, there remains a lack of consensus regarding the long-term prognosis of individuals with DWS [9].

Prognosis is intricately linked to the presence or absence of associated malformations, whether they are genetic, systemic, or neurological in nature, and the severity of each of these coexisting conditions. Therefore, the presence of the number of affected organs is associated with significantly higher neurological morbidity and neonatal mortality [59].

The literature review exposed that there has delineated two distinct prognostic categories within the spectrum of DWM. The first category pertains to cases where the vermis exhibits partial agenesis, while the rest of the brain’s architecture remains intact, often resulting in a life that closely resembles normalcy. Conversely, in cases characterized by significant malformations, encompassing severe dysplasia of the vermis and substantial midline brain anomalies, the prognosis tends to be associated with adverse intellectual and neurological outcomes [60].

Presently, a wide array of diagnostic, therapeutic, and monitoring tools is available for the management of both DWM and DWS. One of the frequently utilized methods in clinical practice involves prenatal assessment of the fetus, including karyotyping and a meticulous examination of the supratentorial midline and vermis [60].

## Conclusion

In conclusion, DWS encompasses a range of cerebellar abnormalities, likely stemming from shared embryological causes, characterized by a large cyst in the posterior fossa along with hydrocephalus and often associated with other CNS anomalies. Prenatal diagnosis typically employs ultrasound and MRI after the 18 th week of gestation, while postnatal confirmation involves imaging focused on cerebellar and posterior fossa abnormalities. Treatment primarily addresses hydrocephalus, often requiring surgical interventions like ventriculoperitoneal shunting or endoscopic third ventriculostomy, with each case requiring a tailored approach based on severity and associated anomalies. Prognosis for DWS varies, heavily influenced by the presence of additional anomalies, with children having concurrent CNS or systemic malformations generally facing worse outcomes. Early diagnosis and intervention are crucial for effective management and improved prognosis.

## Data Availability

No datasets were generated or analysed during the current study.

## Declarations

### Use of artificial intelligence

The authors certify that, at all stages of this study, including writing, data analysis, and information gathering, no artificial intelligence tools or automated technologies were used for the processing or generation of data and results.
